# Supplementary material for: Investigating Los Angeles’ urban roadway network from a biologically-formed perspective
Source: PeerJ. 2020 Jan 13;8:e8238. doi: 10.7717/peerj.8238 (PMC6964692; doi:10.7717/peerj.8238)
Supplement: Data S1 [file peerj-08-8238-s001.zip › Supplemental Material - Raw data files/Specific Base Maps and Distances/Base Map For Trials 11-13.docx]

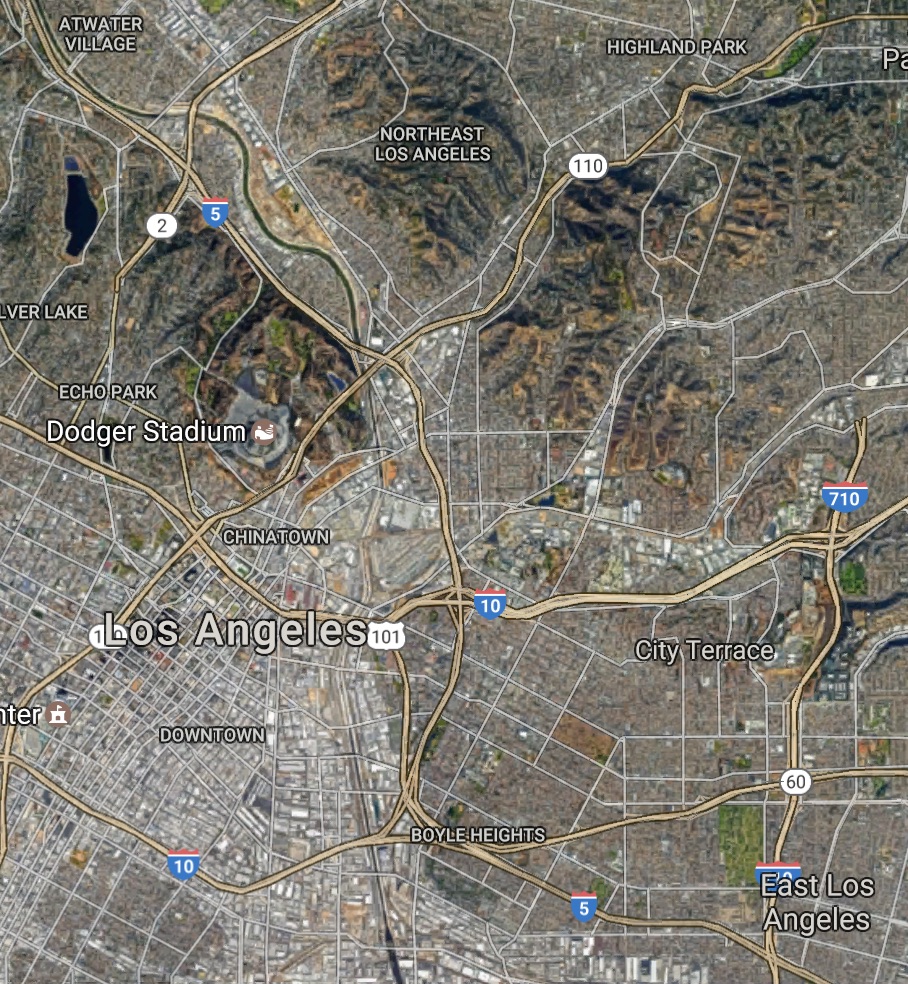


Map data © 2018 Google

G

F

E

C

B

A

D

Oat Flake D Placement: Huntington Dr. S/ El Sereno Ave./ N Eastern Ave.


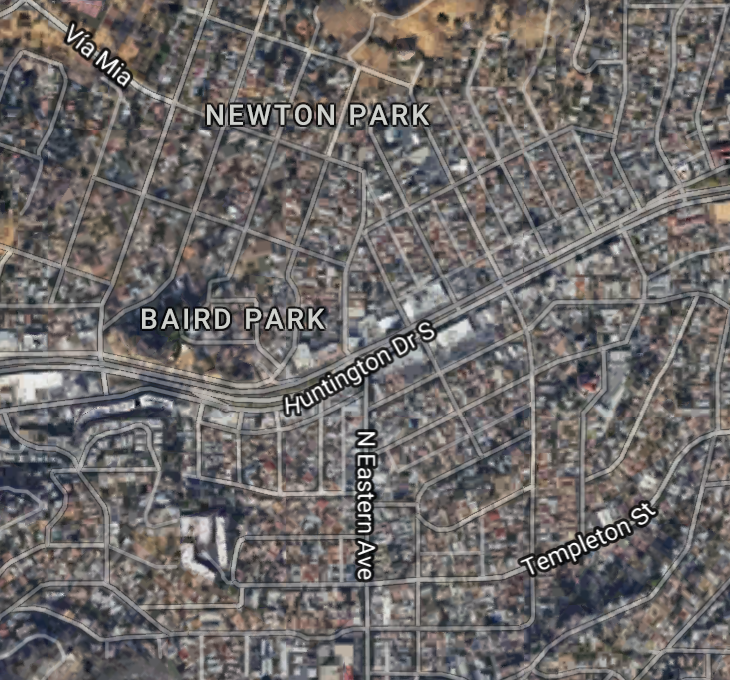


Map data © 2018 Google
