# Supplementary figures and images for: Investigating Los Angeles’ urban roadway network from a biologically-formed perspective
Source: PeerJ. 2020 Jan 13;8:e8238. doi: 10.7717/peerj.8238 (PMC6964692; doi:10.7717/peerj.8238)

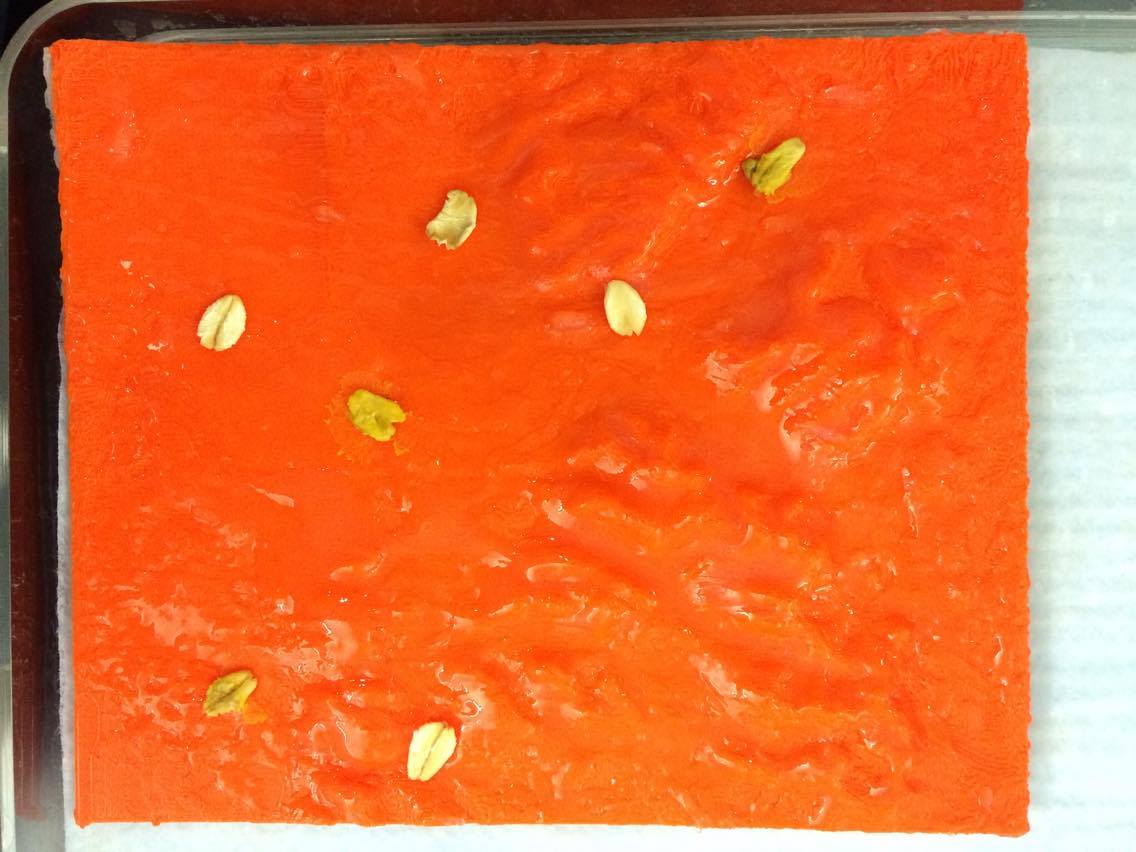

Supplement: Data S1 [file peerj-08-8238-s001.zip › Supplemental Material - Raw data files/Trial 1 9_26-9_29_17/DTLA #2 9_26_17/DTLA #2 9_26_17 10pm.jpg]

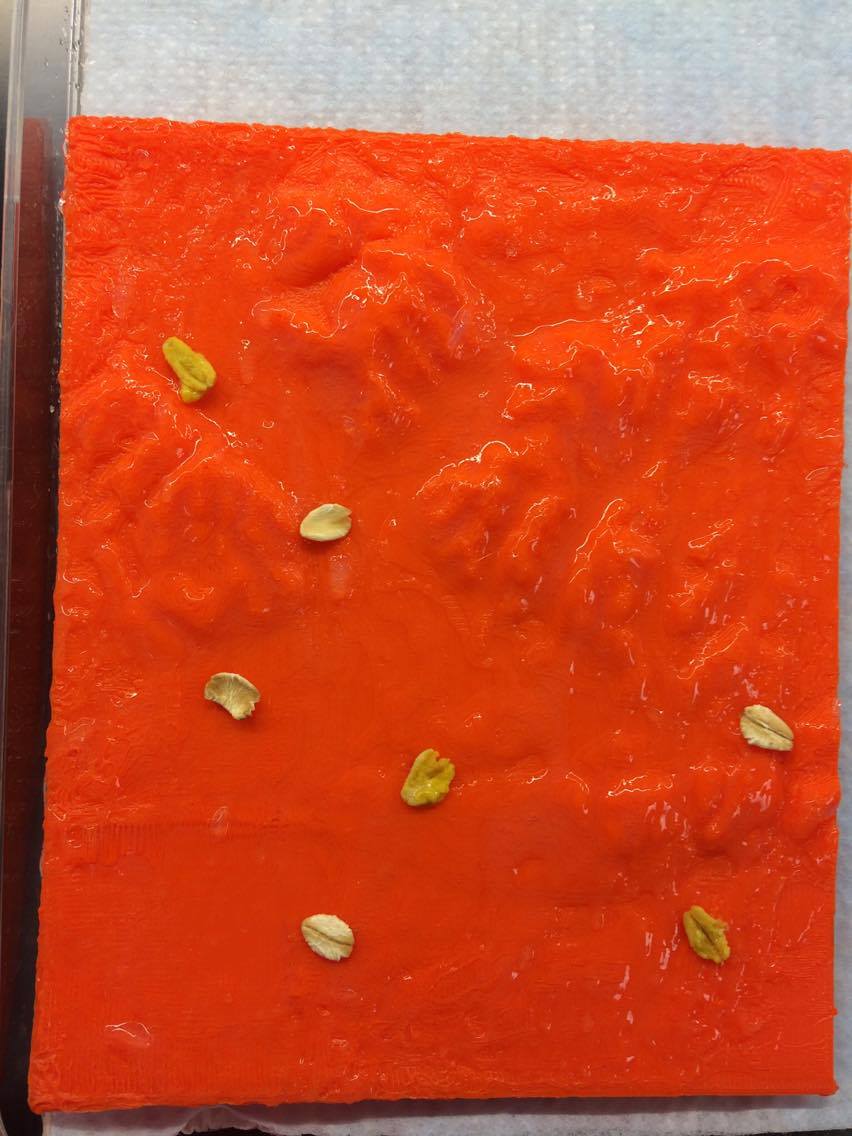

Supplement: Data S1 [file peerj-08-8238-s001.zip › Supplemental Material - Raw data files/Trial 1 9_26-9_29_17/DTLA #2 9_26_17/DTLA #2 9_26_17 2pm.jpg]

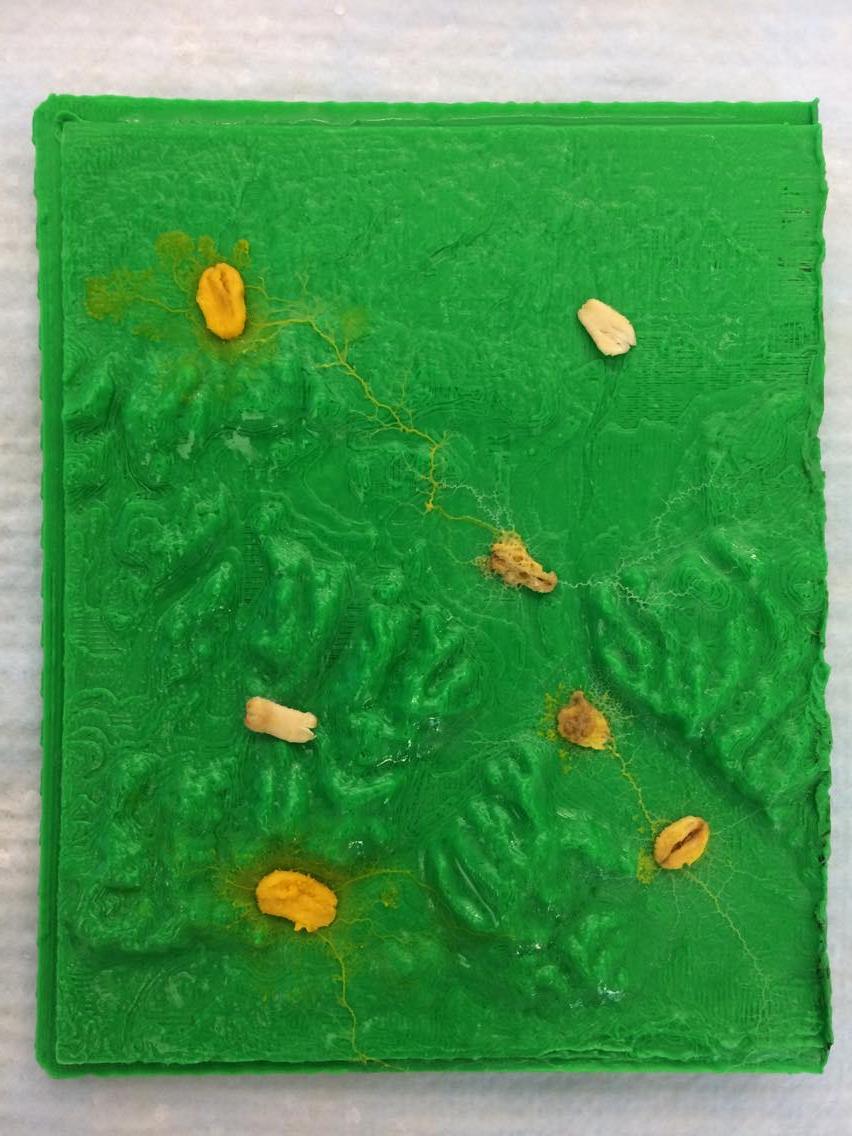

Supplement: Data S1 [file peerj-08-8238-s001.zip › Supplemental Material - Raw data files/Unedited Slime mold DTLA exploration picture/DTLA 14.jpg]

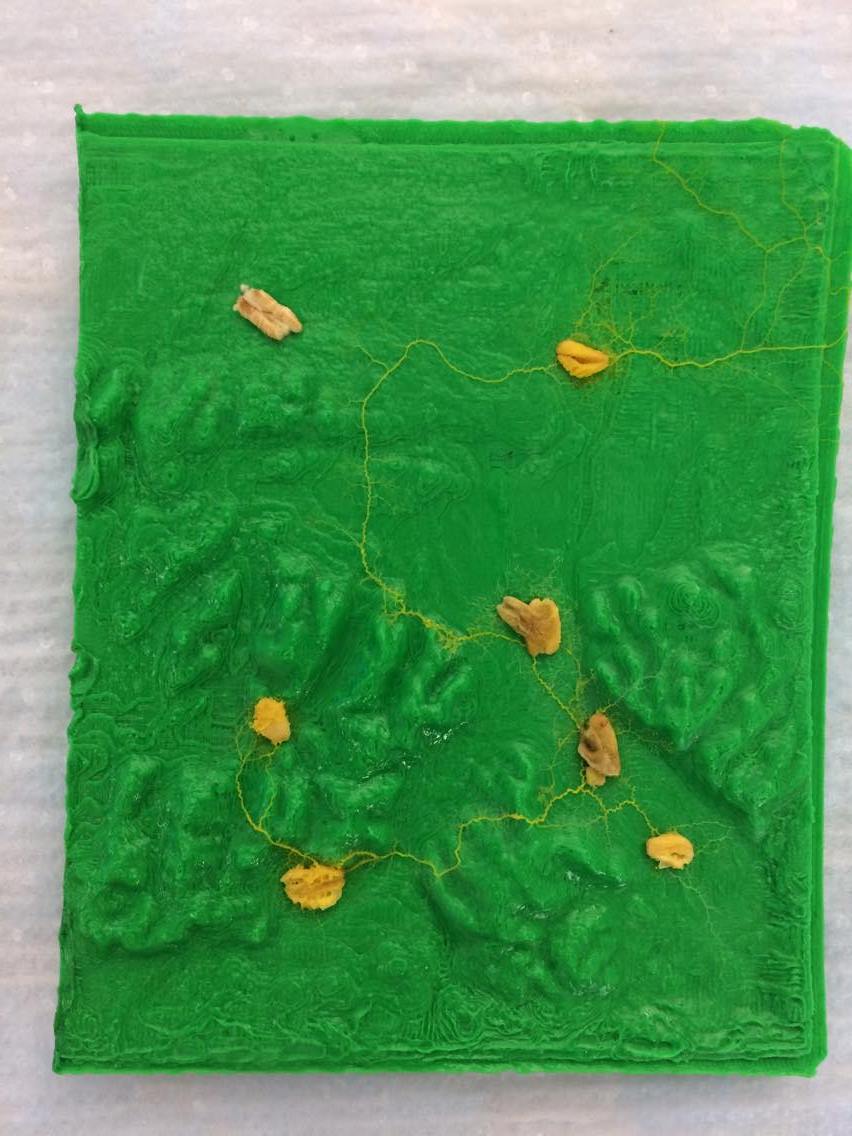

Supplement: Data S1 [file peerj-08-8238-s001.zip › Supplemental Material - Raw data files/Unedited Slime mold DTLA exploration picture/DTLA 15.jpg]

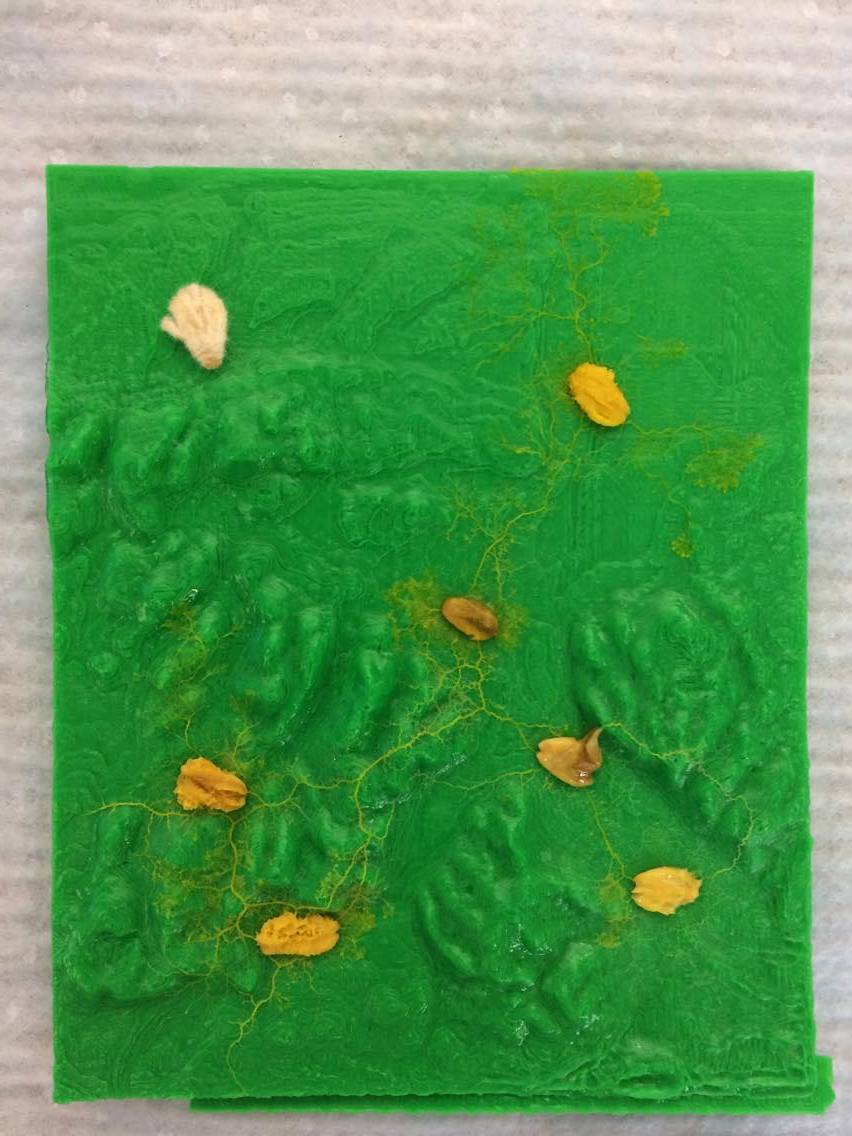

Supplement: Data S1 [file peerj-08-8238-s001.zip › Supplemental Material - Raw data files/Unedited Slime mold DTLA exploration picture/DTLA 16.jpg]

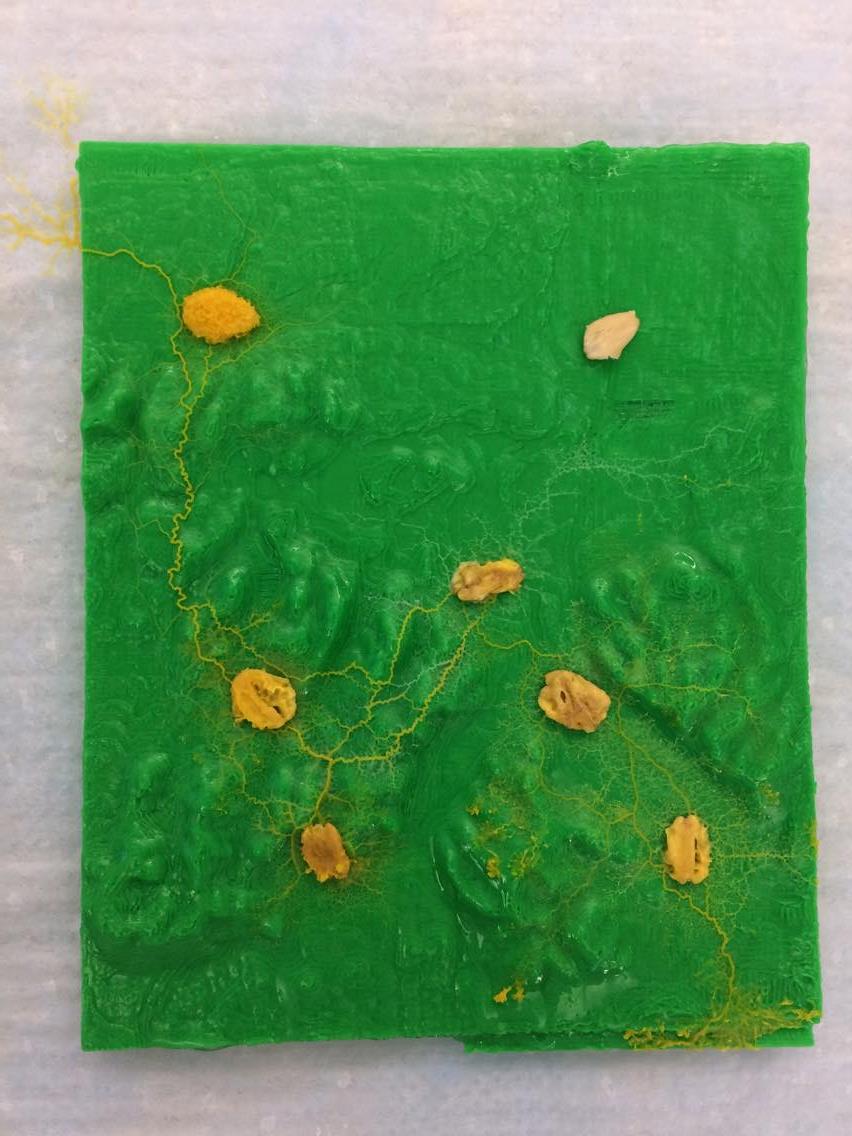

Supplement: Data S1 [file peerj-08-8238-s001.zip › Supplemental Material - Raw data files/Unedited Slime mold DTLA exploration picture/DTLA 17.jpg]

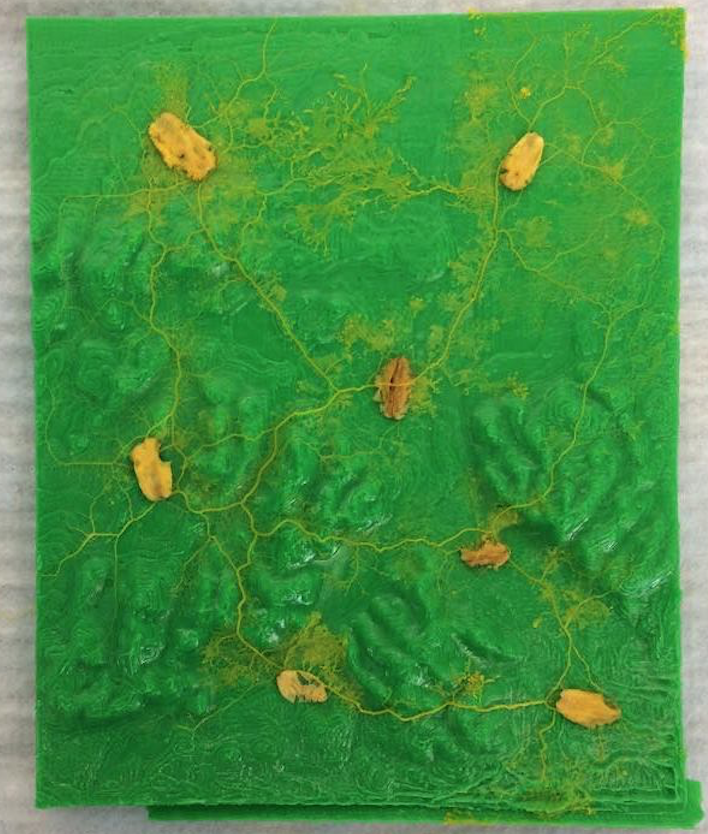

Supplement: Data S1 [file peerj-08-8238-s001.zip › Supplemental Material - Raw data files/Unedited Slime mold DTLA exploration picture/DTLA1.png]

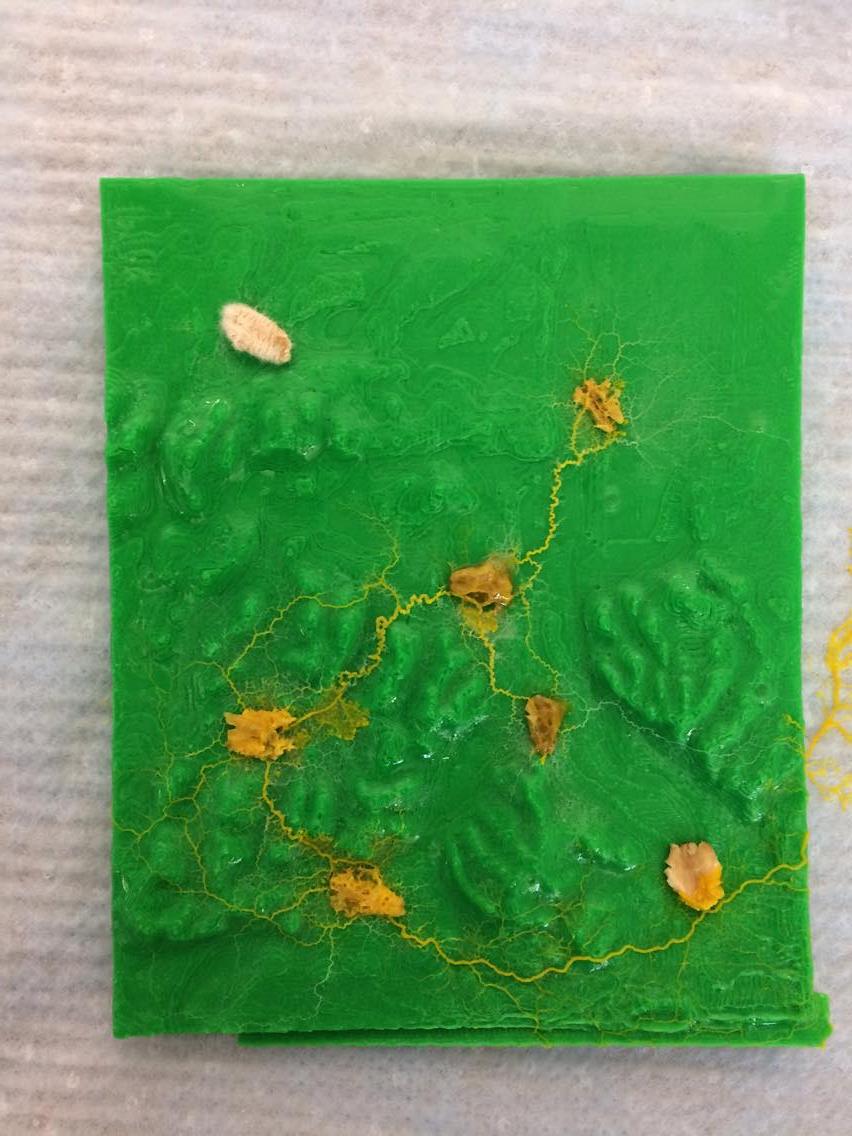

Supplement: Data S1 [file peerj-08-8238-s001.zip › Supplemental Material - Raw data files/Unedited Slime mold DTLA exploration picture/DTLA10.jpg]

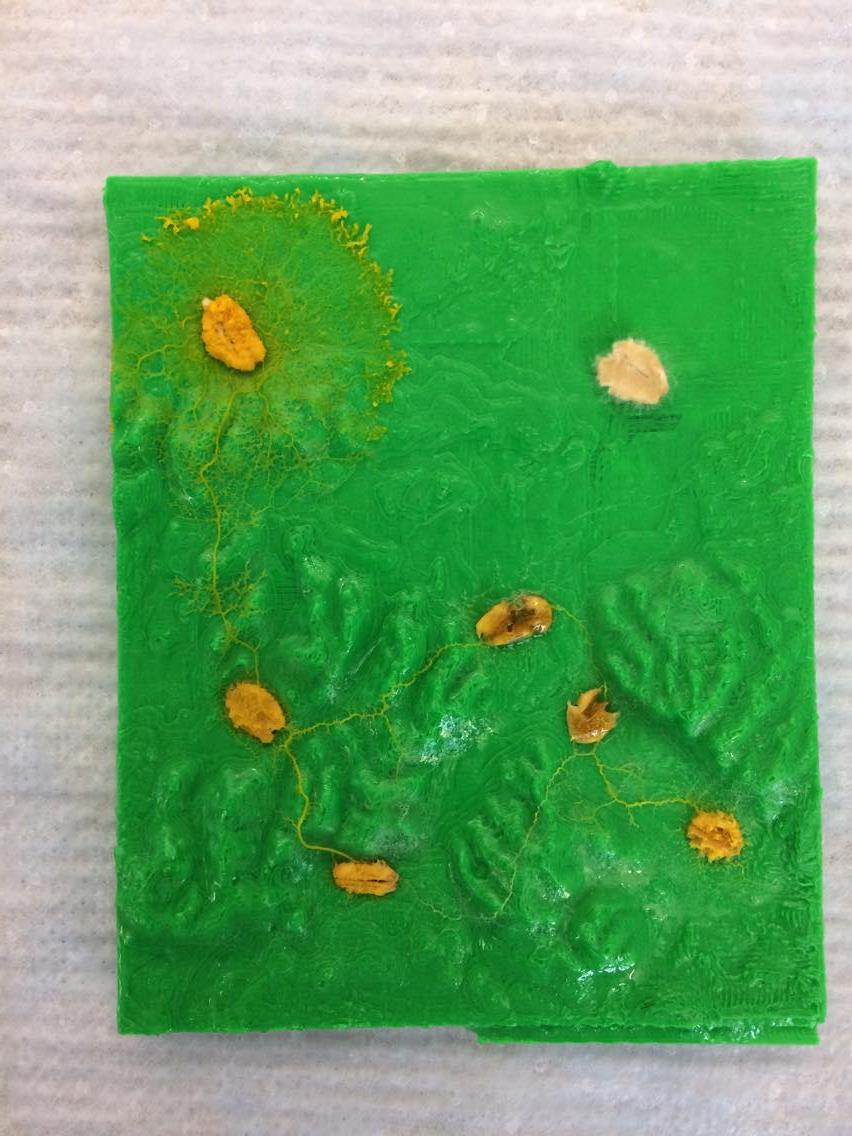

Supplement: Data S1 [file peerj-08-8238-s001.zip › Supplemental Material - Raw data files/Unedited Slime mold DTLA exploration picture/DTLA11.jpg]

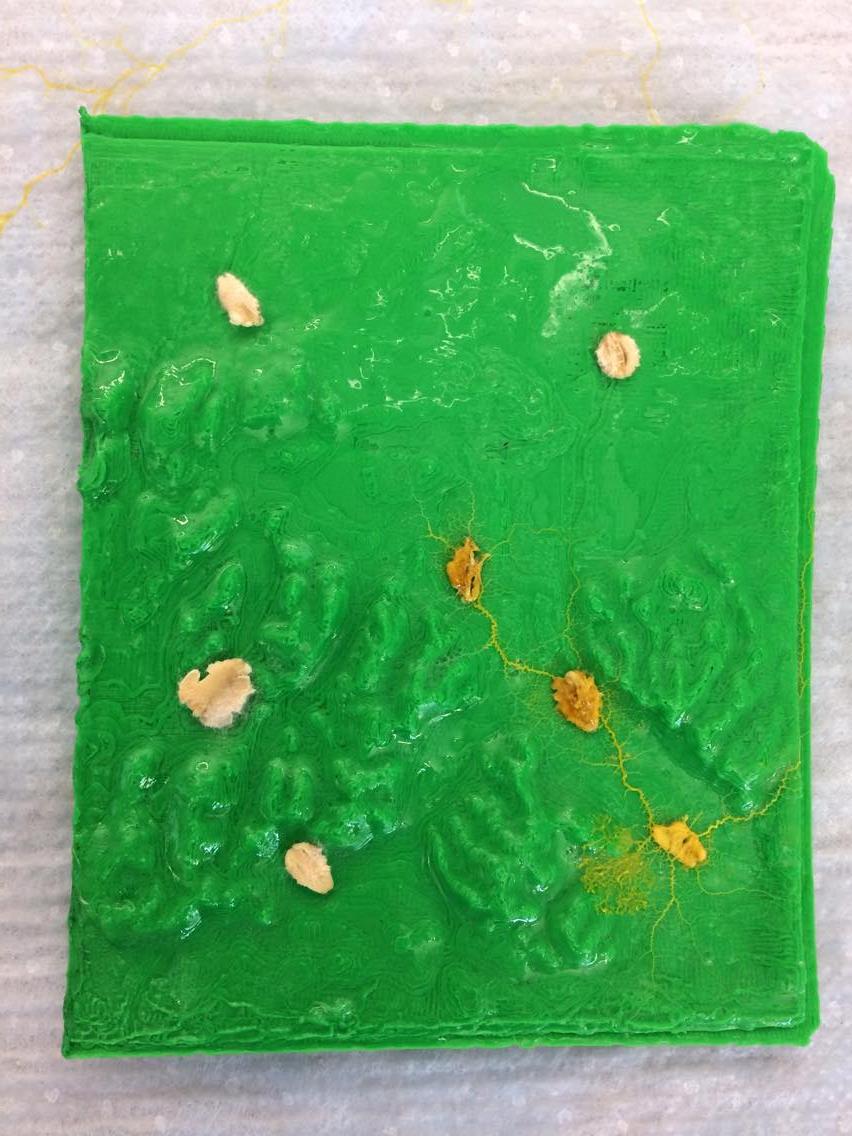

Supplement: Data S1 [file peerj-08-8238-s001.zip › Supplemental Material - Raw data files/Unedited Slime mold DTLA exploration picture/DTLA12.jpg]

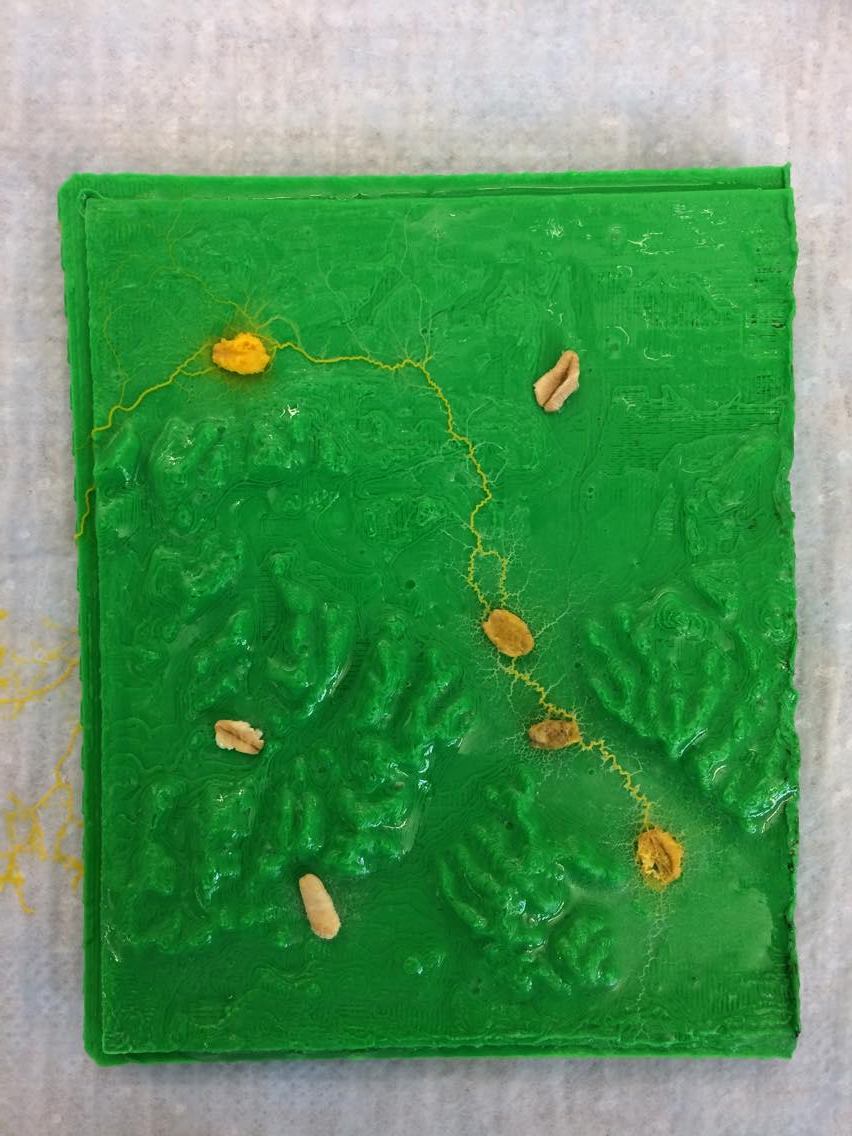

Supplement: Data S1 [file peerj-08-8238-s001.zip › Supplemental Material - Raw data files/Unedited Slime mold DTLA exploration picture/DTLA13.jpg]

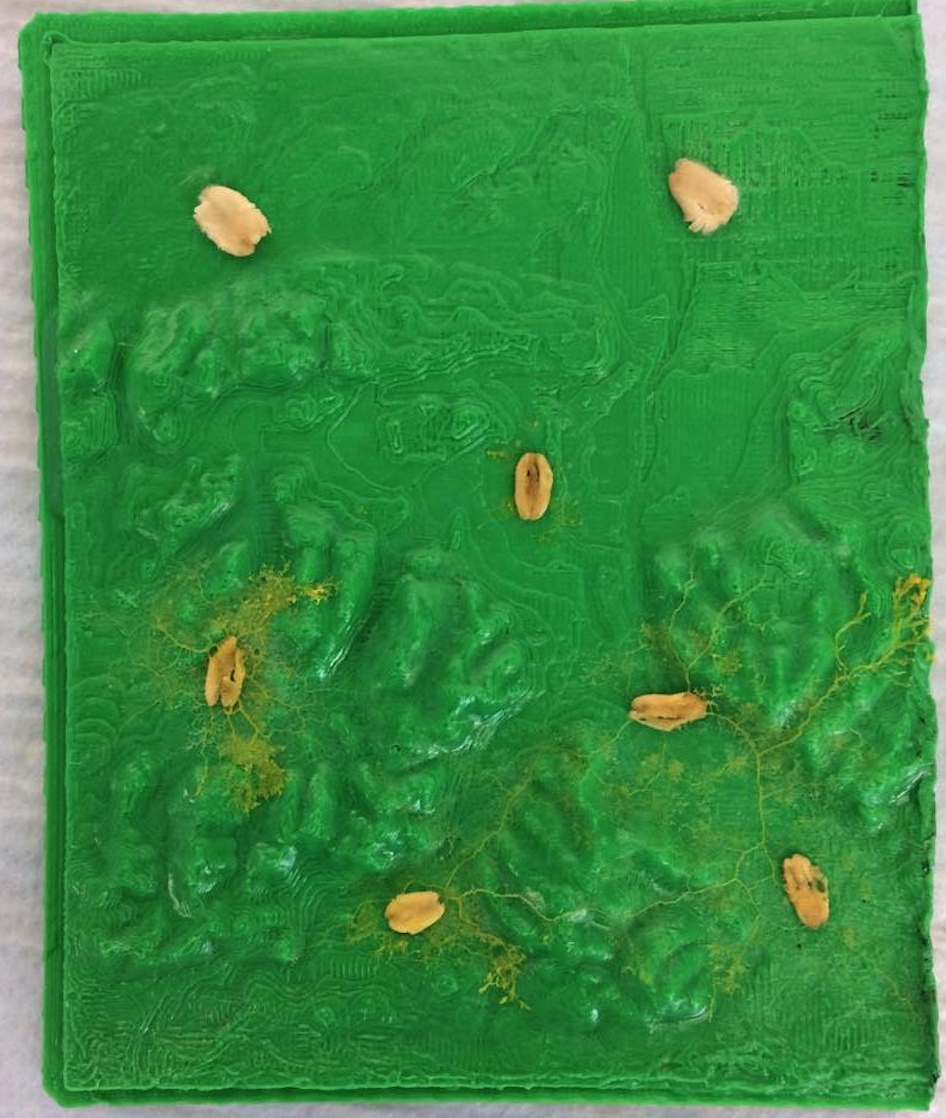

Supplement: Data S1 [file peerj-08-8238-s001.zip › Supplemental Material - Raw data files/Unedited Slime mold DTLA exploration picture/DTLA2.png]

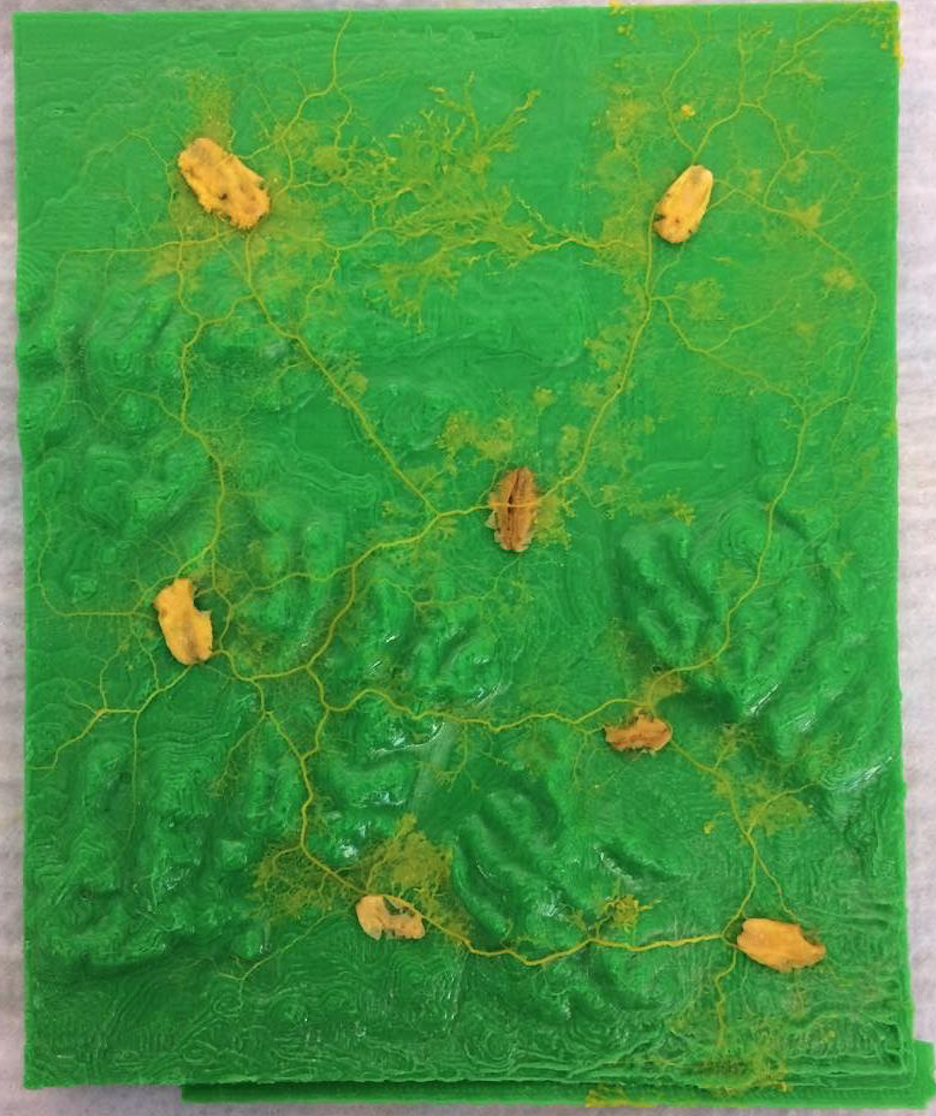

Supplement: Data S1 [file peerj-08-8238-s001.zip › Supplemental Material - Raw data files/Unedited Slime mold DTLA exploration picture/DTLA3.png]

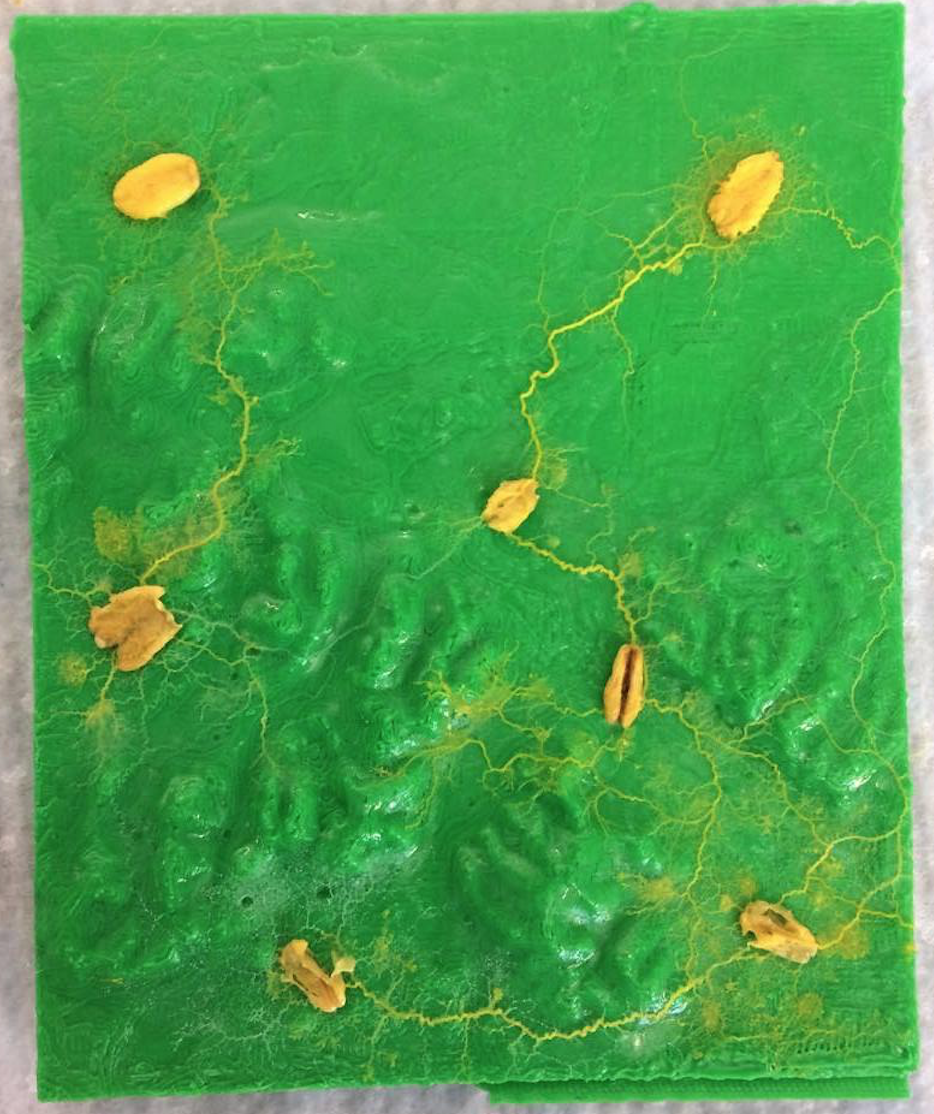

Supplement: Data S1 [file peerj-08-8238-s001.zip › Supplemental Material - Raw data files/Unedited Slime mold DTLA exploration picture/DTLA4.png]

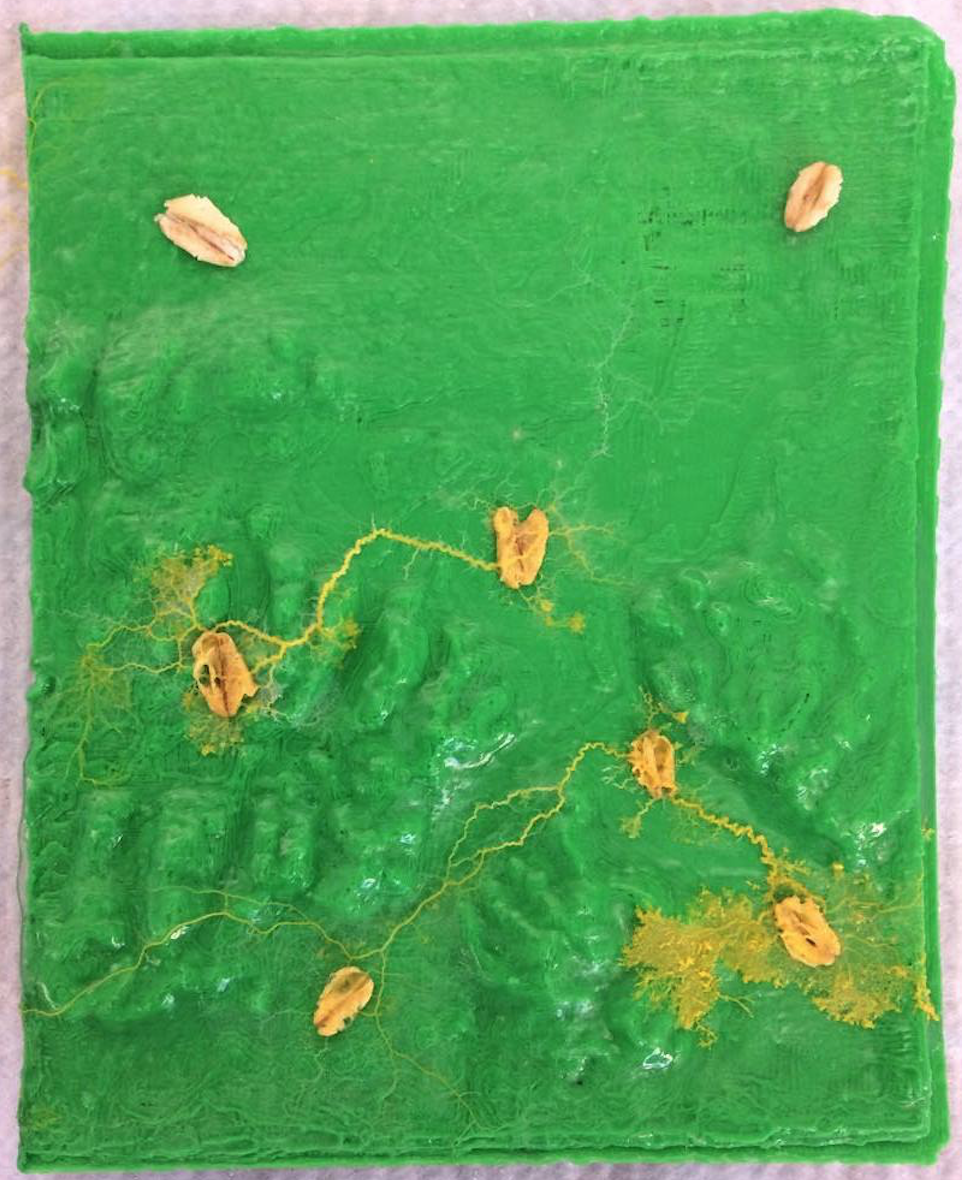

Supplement: Data S1 [file peerj-08-8238-s001.zip › Supplemental Material - Raw data files/Unedited Slime mold DTLA exploration picture/DTLA5.png]

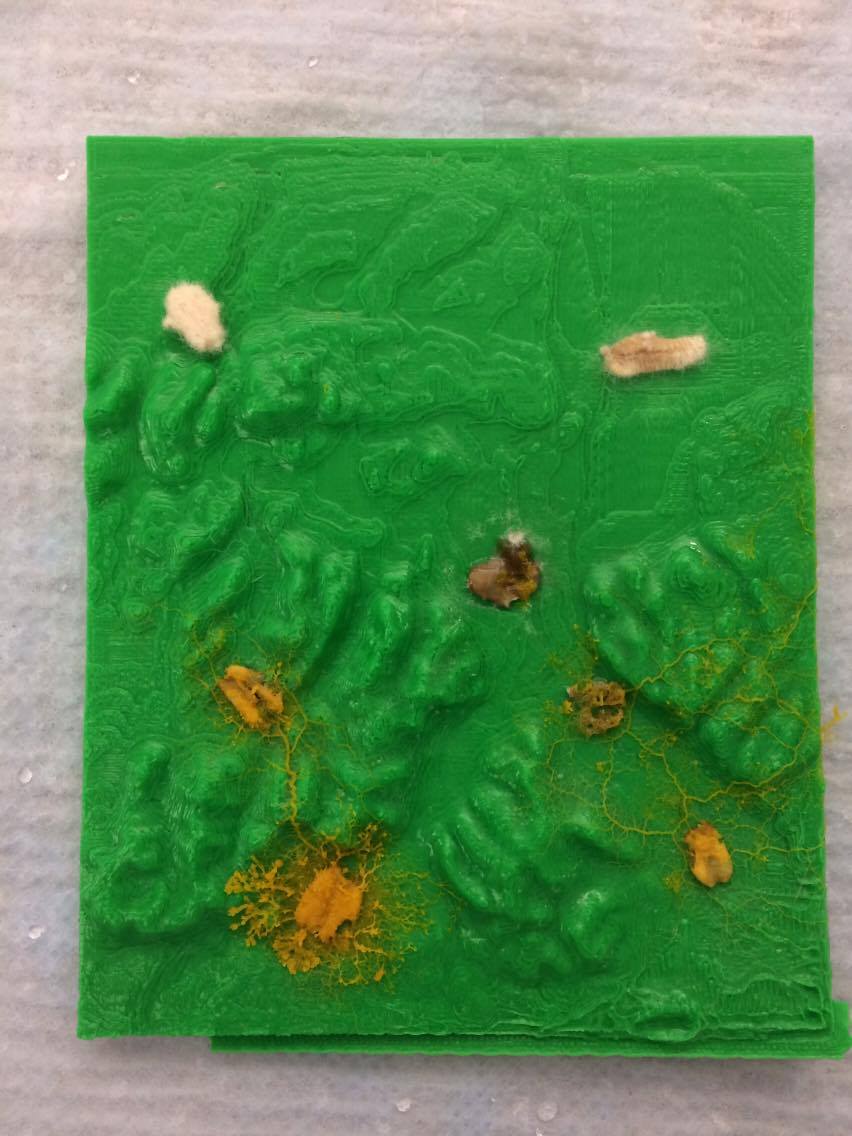

Supplement: Data S1 [file peerj-08-8238-s001.zip › Supplemental Material - Raw data files/Unedited Slime mold DTLA exploration picture/DTLA6.jpg]

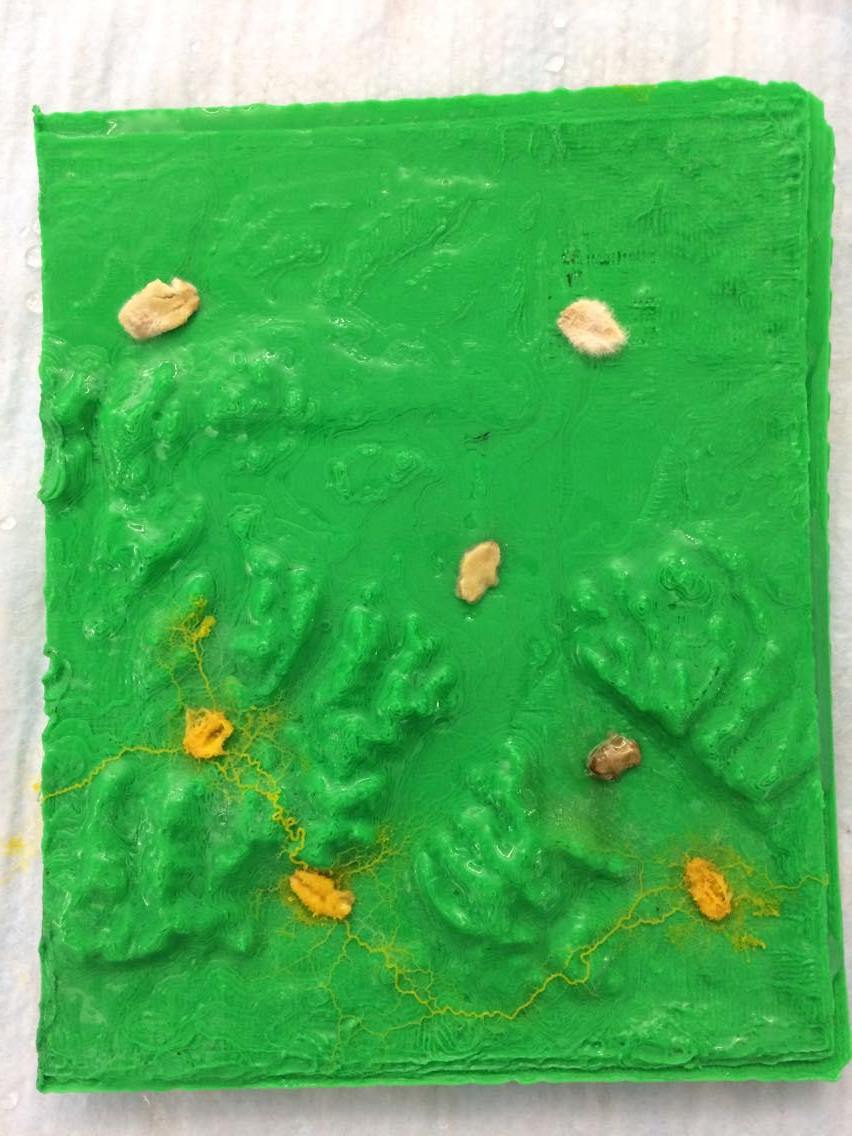

Supplement: Data S1 [file peerj-08-8238-s001.zip › Supplemental Material - Raw data files/Unedited Slime mold DTLA exploration picture/DTLA7.jpg]

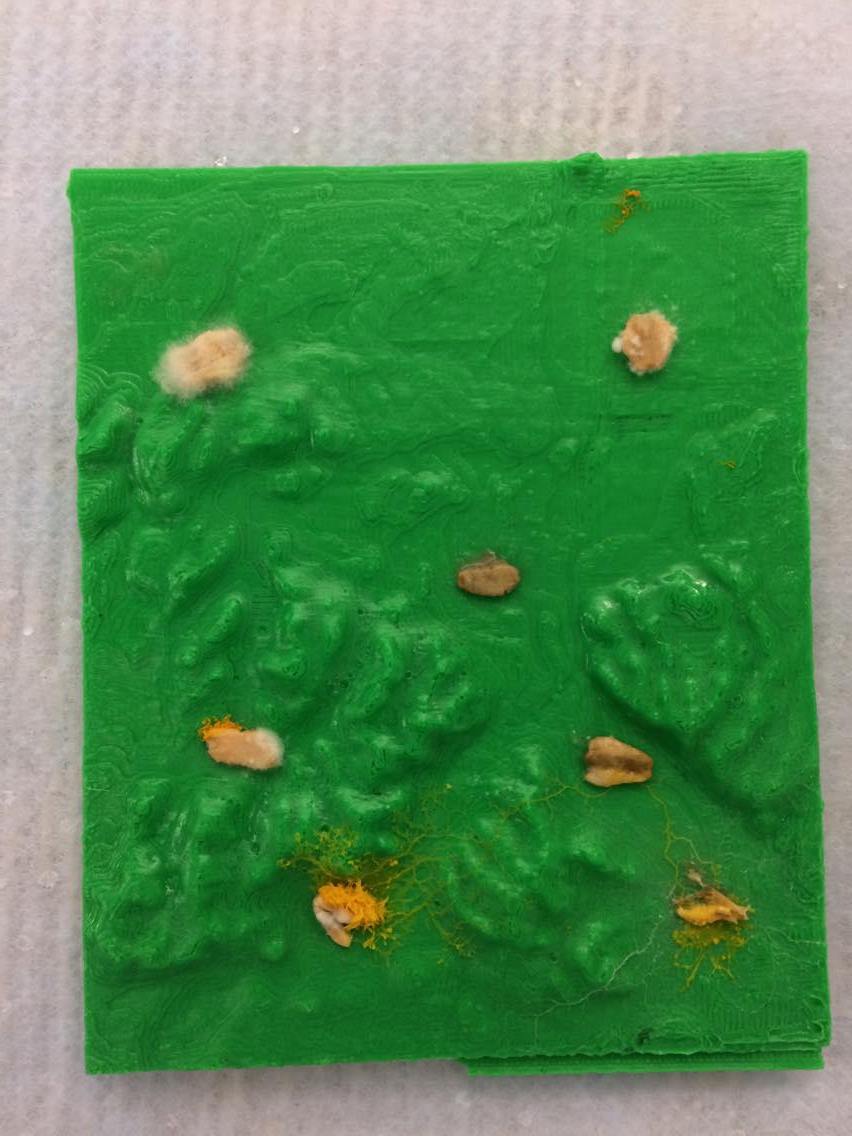

Supplement: Data S1 [file peerj-08-8238-s001.zip › Supplemental Material - Raw data files/Unedited Slime mold DTLA exploration picture/DTLA8.jpg]

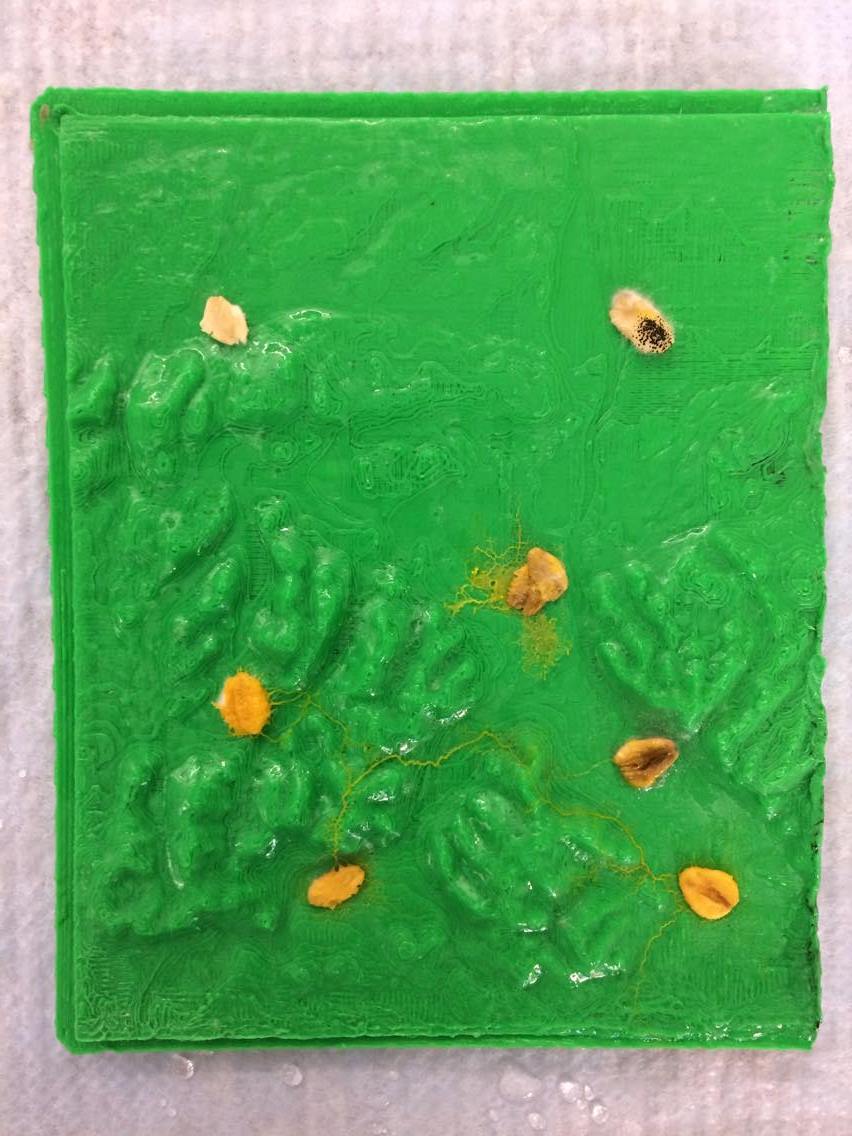

Supplement: Data S1 [file peerj-08-8238-s001.zip › Supplemental Material - Raw data files/Unedited Slime mold DTLA exploration picture/DTLA9.jpg]
